# Influence of Cr Substitution and Temperature on Hierarchical Phase Decomposition in the AlCoFeNi High Entropy Alloy

**DOI:** 10.1038/s41598-018-33922-w

**Published:** 2018-10-22

**Authors:** V. Chaudhary, B. Gwalani, V. Soni, R. V. Ramanujan, R. Banerjee

**Affiliations:** 1 School of Materials Science and Engineering, Nanyang Technological University, Singapore, 639798 Singapore; 2 Department of Materials Science and Engineering, University of North Texas, Denton, TX 76201 USA; 3 Singapore-HUJ Alliance for Research and Enterprise (SHARE), Nanomaterials for Energy and Energy-Water Nexus (NEW), Campus for Research Excellence and Technological Enterprise (CREATE), Singapore, 138602 Singapore

## Abstract

While the AlCoFeNi high entropy alloy exhibits a single ordered B2 phase at high temperature, both the substitution of ferromagnetic Co with antiferromagnetic Cr, and lower annealing temperatures lead to a tendency for this system to decompose into a two-phase mixture of ordered B2 and disordered BCC solid solution. The length scale of this decomposition is determined by the combination of composition and annealing temperature, as demonstrated in this investigation by comparing and contrasting AlCoFeNi with the AlCo_0.5_Cr_0.5_FeNi alloy. The resulting phase stability has been rationalized based on solution thermodynamic predictions. Additionally, it is shown that replacement of Co by Cr in the AlCoFeNi alloy resulted in a substantial reduction in saturation magnetization and increase in coercivity. The microhardness is also strongly influenced by the composition and the length scale of B2 + BCC decomposition in these high entropy alloys.

## Introduction

High-entropy alloys (HEAs) have attracted a lot of attention in recent years, due to the unique combinations of structural, physical and chemical properties of these alloys^1–3^. HEAs usually form simple structures such as body-centered cubic (BCC) (e.g. TaNbHfZrTi^4^, NbTaVWZr^5^ etc.) or face-centered cubic (FCC) (e.g., CoCrCuFeNi^6^, CoCrFeMnNi^7^ etc.) solid solutions. The large number of the alloying elements in near-equiatomic proportions result in high configurational entropy in these solid solutions. However, these alloys can decompose to multiple phases upon changing the composition or annealing at different temperatures or a combination of both^8–10^. Such a phase decomposition is often the result of the complex interplay of configurational entropy of mixing with the enthalpies of mixing of the constituent elements. Additionally, such multiphase microstructures are more likely to exhibit the desired properties for real engineering applications. Hence, tuning the composition and fractions of phases is critical for achieving the desired properties^10,11^.

Recently, it has been reported that AlCoCrFeNi based HEAs exhibit high hardness, good corrosion resistance, high yield strength and promising magnetic properties^1,11–15^. A systematic investigation on a compositionally graded AlCo*x*Cr_1−*x*_FeNi sample, processed by laser engineered net shape (LENS) technology^13^, revealed that all the compositions within this graded sample exhibited either a single B2 phase or a two-phase BCC + B2 microstructure. A separate study reported that annealing the AlCoCrFeNi HEA at 600 °C, exhibited a near single B2 phase microstructure, while the same alloy annealed in the temperature range of 800 to 1200 °C exhibited FCC precipitates in a B2 matrix^15^. While the influence of Al content on the phases and microstructures, in the Al_x_CoCrFeNi class of HEAs has been extensively investigated^10,16,17^, there is rather limited knowledge regarding the composition and temperature dependent microstructure and properties in the AlCo_x_Cr_1−x_FeNi system^13^. Therefore, the role of Cr, an anti-ferromagnetic element, on the microstructural evolution and properties (magnetic and mechanical) of AlCo_x_Cr_1−x_FeNi type HEAs, needs further investigation and forms the basis of the present study. This study focuses on two representative compositions, i.e., AlCoFeNi and AlCo_0.5_Cr_0.5_FeNi processed by conventional melt technology and subsequently annealed at specific temperatures/times to determine the phase stability as a function of composition and temperature. The complex phase decomposition in these alloys, over multiple length scales, has been analyzed in detail by coupling multiple microscopy techniques, including scanning electron microscopy (SEM) (including electron backscatter diffraction (EBSD)), transmission electron microscopy (TEM) and atom probe tomography (APT). The experimentally observed phase decomposition has been rationalized using solution thermodynamic models (CALPHAD approach). The resultant mechanical (microhardness) and magnetic properties of these alloys have also been characterized as a function of composition and annealing temperature.

## Experimental Details

AlCoxCr_1−x_FeNi (x = 0.5 and 1) HEAs were prepared by arc melting of elemental Al, Co, Cr, Fe and Ni pellets (99.9% purity) under argon atmosphere. Alloys were repeatedly melted to achieve good chemical homogeneity. The resulting ingots were annealed at 600 °C and 1000 °C for 15 h under argon and subsequently quenched in water. The composition and microstructures were characterized by a scanning electron microscope (SEM), using a FEI Nova-NanoSEM 230™ coupled with an energy dispersive x-ray spectrum analyzer (SEM–EDS). Site-specific TEM samples were prepared using a FEI Nova 200 dual beam focused ion beam (FIB), and these samples were characterized in a FEI Tecnai F20™ FEG TEM operating at 200 kV. Standard lift-out technique was used for for Atom Probe Tomography (APT) sample preparation using a FIB. APT experiments were conducted on a CAMECA local electrode atom probe 3000X HR instrument under voltage mode. The target pulse fraction was 0.2 and the temperature of 40–50 K was maintained while the experiments. APT data reconstruction and analysis was carried out using CAMECA IVAS® 3.6.8 software. The Kf and ICF (image compression factor) used for the reconstruction of the tips were 3.3 and 1.650, respectively. The tips are reconstructed using initial radius of 30 nm and shank half angle of 20°, which is based on the SEM examination of the tips taken after the final milling.

## Results and Discussion

### Microstructure of AlCoFeNi after isothermal annealing at 600 °C and 1000 °C

**Figure 1:**
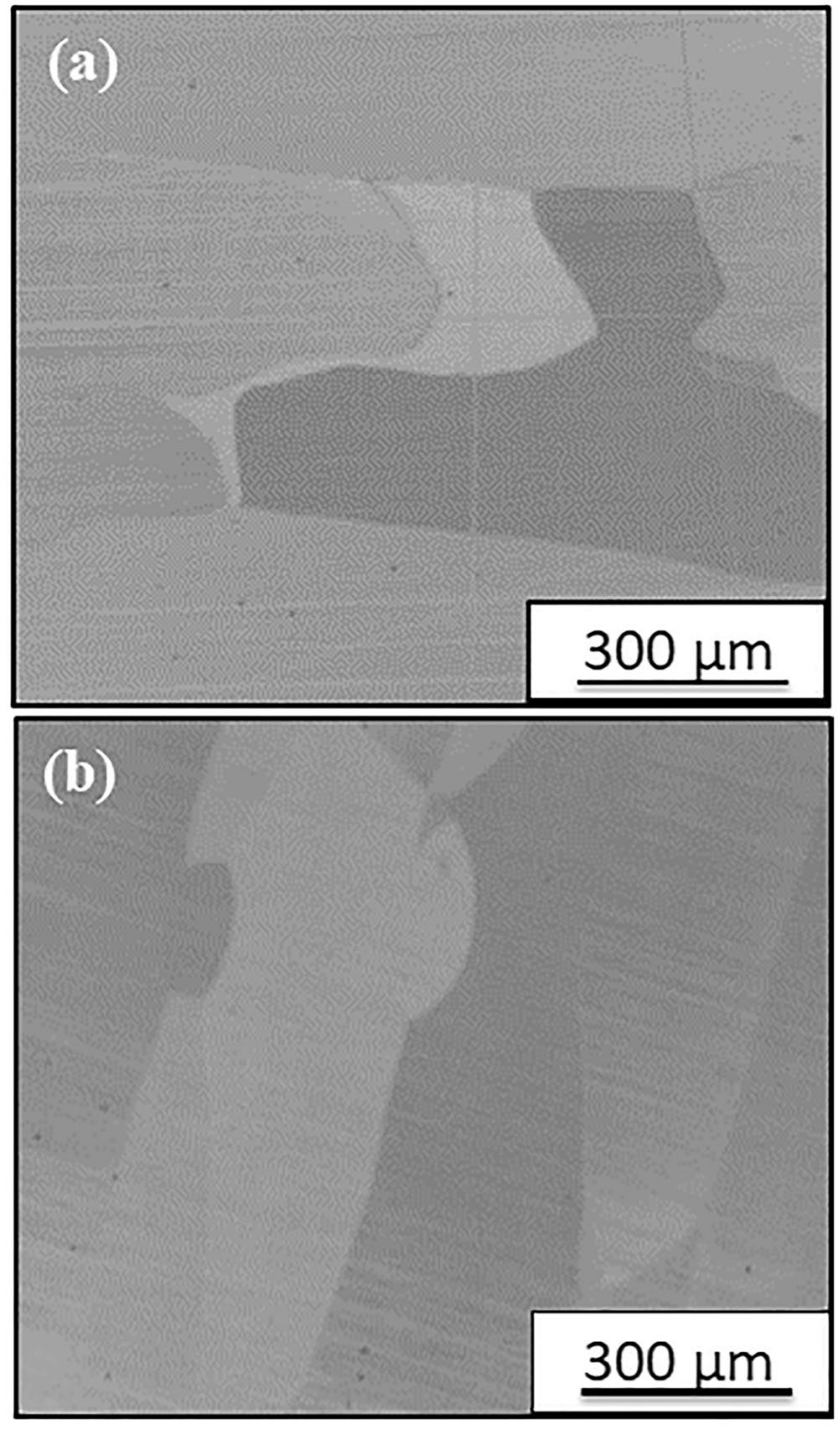
Back-scattered SEM image of AlCoFeNi specimens annealed for 15 h at (**a**) 1000 °C and (**b**) 600 °C.

Samples of the AlCoFeNi alloy were isothermally annealed at either 600 **°**C or 1000 **°**C for 15 h. Figure 1 shows the back-scatter SEM images of these annealed samples. The samples exhibited a polycrystalline microstructure consisting of large grains. Based on these images the microstructure for both samples appears to be a single phase since no phase separation or second phase precipitation is evident from these SEM images. However, it should be recognized that finer scale second phase precipitates or phase separation may be present in these samples but not revealed at this coarse length scale of imaging. Hence, more detailed investigations have been carried out using TEM and APT.

#### TEM and APT investigation of AlCoFeNi (1000 °C/15 h)

**Figure 2:**
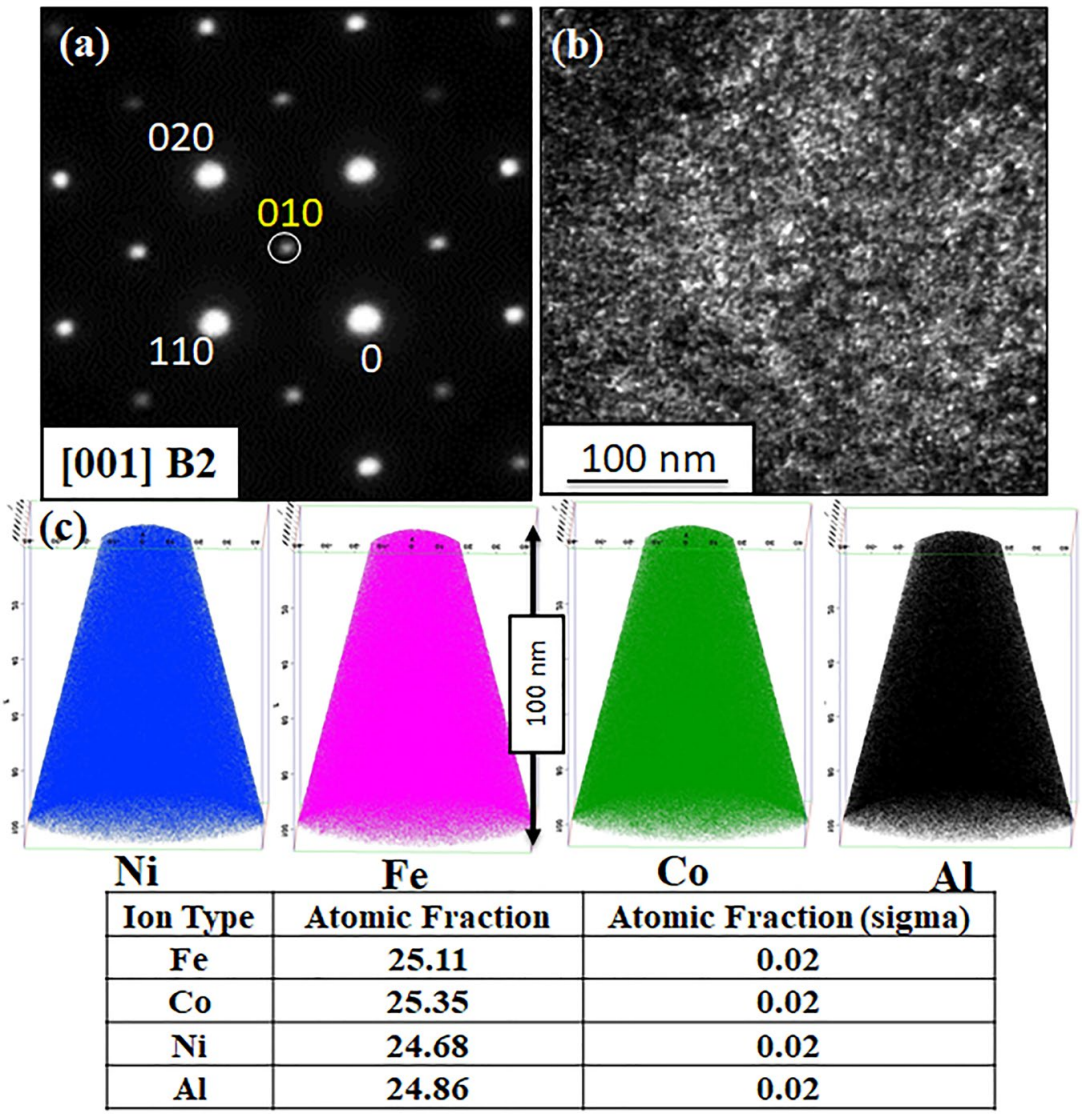
TEM and APT results of AlCoFeNi alloy annealed for 15 h at 1000 °C. (**a**) Selected area diffraction (SAD) pattern from [001]_B2_ ZA (**b**) corresponding dark-field TEM image taken using the encircled superlattice spot (**c**) ionic 3D reconstructions of Ni, Fe, Co and Al atoms, respectively.

Figure 2(a) shows the selected area diffraction pattern (SADP) from the [001]_BCC_ zone axis from the AlCoFeNi alloy annealed at 1000 **°**C for 15 h. The superlattice spots at {100} positions (one such spot is highlighted by a white circle and labeled as <010> in Fig. 2(a)) clearly proves the presence of an ordered BCC (B2) phase. A dark field TEM (DFTEM) image recorded using the <010> spot labeled in Fig. 2(a) is shown in Fig. 2(b). While this DFTEM image does exhibit some contrast with particle-like features, it is not possible to definitively attribute the contrast to precipitates, rather than simply strain contrast within the continuous B2 matrix. Therefore, atom probe tomography (APT) was performed to investigate the possibility of phase separation within the B2 matrix. Individual ionic/elemental reconstructions of Ni, Fe, Co and Al atoms (Fig. 2(c)) reveal a homogenous elemental distribution. The atomic fraction of Ni, Fe, Co and Al are tabulated in Fig. 2(c). Thus, these AlCoFeNi samples consist of a single phase with a B2 crystal structure.

#### TEM and APT investigation of AlCoFeNi (600 °C/15 h)

**Figure 3:**
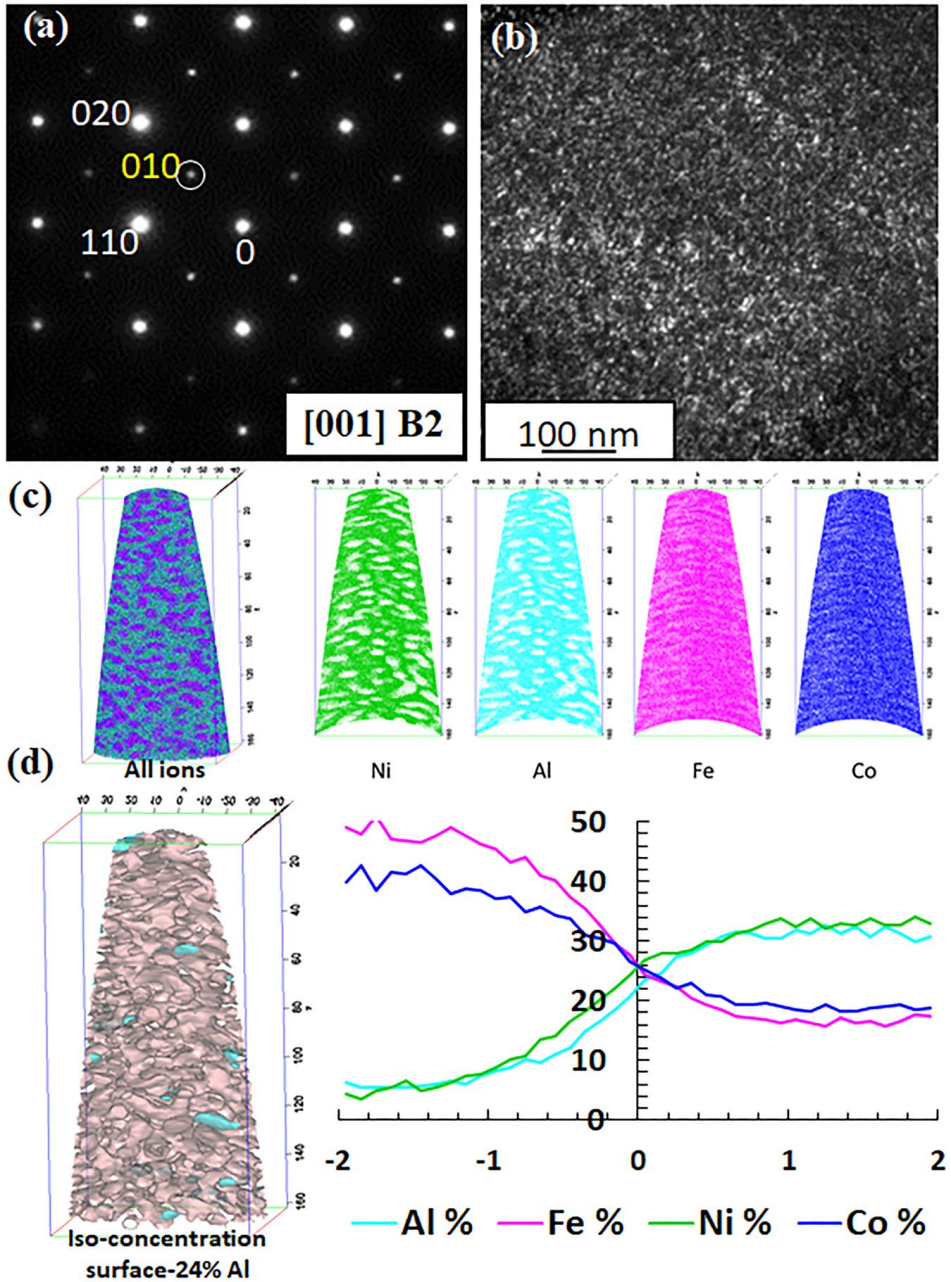
TEM and APT results of AlCoFeNi alloy annealed for 15 h at 600 °C. (**a**) Selected area diffraction (SAD) pattern from [001]_B2_ ZA (**b**) dark-field TEM image from the encircled super-lattice spot (**c**) ionic 3D reconstructions of all ions, Ni, Al, Fe and Co atoms, respectively (**d**) Iso-concentration surfaces are used to create interfaces between the BCC and B2 regions as shown in left side. A proximity histogram is created to show the compositional partitioning across the interfaces (right side).

The TEM results after annealing the AlCoFeNi alloy at 600 °C for 15 h are shown in Fig. 3. Figure 3(a) shows the SADP from the [001] zone axis of the BCC lattice. Similar to the previous condition, superlattice spots corresponding to the B2phase are evident. Figure 3(b) shows the DFTEM generated from a <010> superlattice spot. This DFTEM image appears to be nearly identical to the one observed in case of the 1000 °C/15 h annealed sample, shown in Fig. 2(b). Therefore, it is not possible to establish phase separation within the B2 matrix based on this dark-field image. Figure 3(c) and (d) show APT results from the 600 °C/15 h annealed AlCoFeNi alloy. 3D reconstructions of ions corresponding to all the elements, Ni, Fe, Co and Al have been plotted in Fig. 3(c). Fine scale precipitate-like regions rich in Fe and Co were observed, the matrix is rich in Al and Ni. The compositional partitioning observed in the APT reconstruction was analyzed by constructing iso-concentration surfaces (isosurfaces) along 24.5 at% Al. Interfaces between the two regions were constructed (Fig. 3(d)) and a proximity histogram, delineating the two phases,and showing elemental partitioning is also shown in Fig. 3(d) (right side). From the APT and TEM results, the two phases can be characterized as the Al-Ni rich B2 matrix interspersed with fine scale precipitates of a Fe-Co rich BCC phase. The APT results revealed that the average compositions of the BCC phase is 6Al-40Co-49Fe-5Ni (at%), while that of the B2 phase was determined to be 32Al-18Co-17Fe-33Ni (at%).

### Effect of partial Co replacement by Cr: Microstructure of AlCo_0.5_Cr_0.5_FeNi alloy after isothermal annealing at 1000 °C and 600 °C

#### SEM and EDS-EBSD investigation of AlCo_0.5_Cr_0.5_FeNi

**Figure 4:**
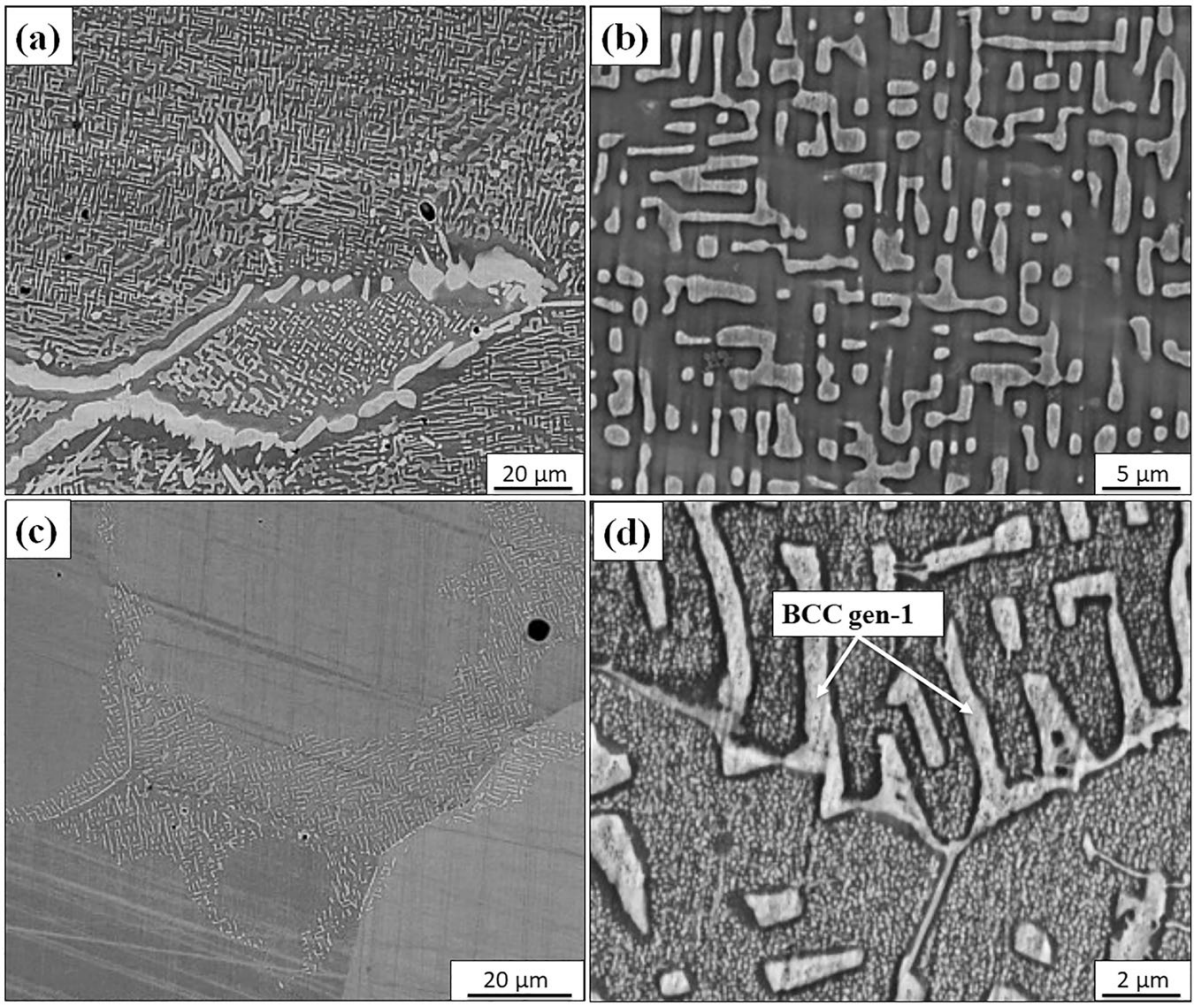
Back-scattered SEM image of AlCo_0.5_Cr_0.5_FeNi specimens annealed for 15 h at (**a**) 1000 °C and (**c**) 600 °C, corresponding high magnification images are shown in (**b**) and (**d**), respectively.

**Figure 5:**
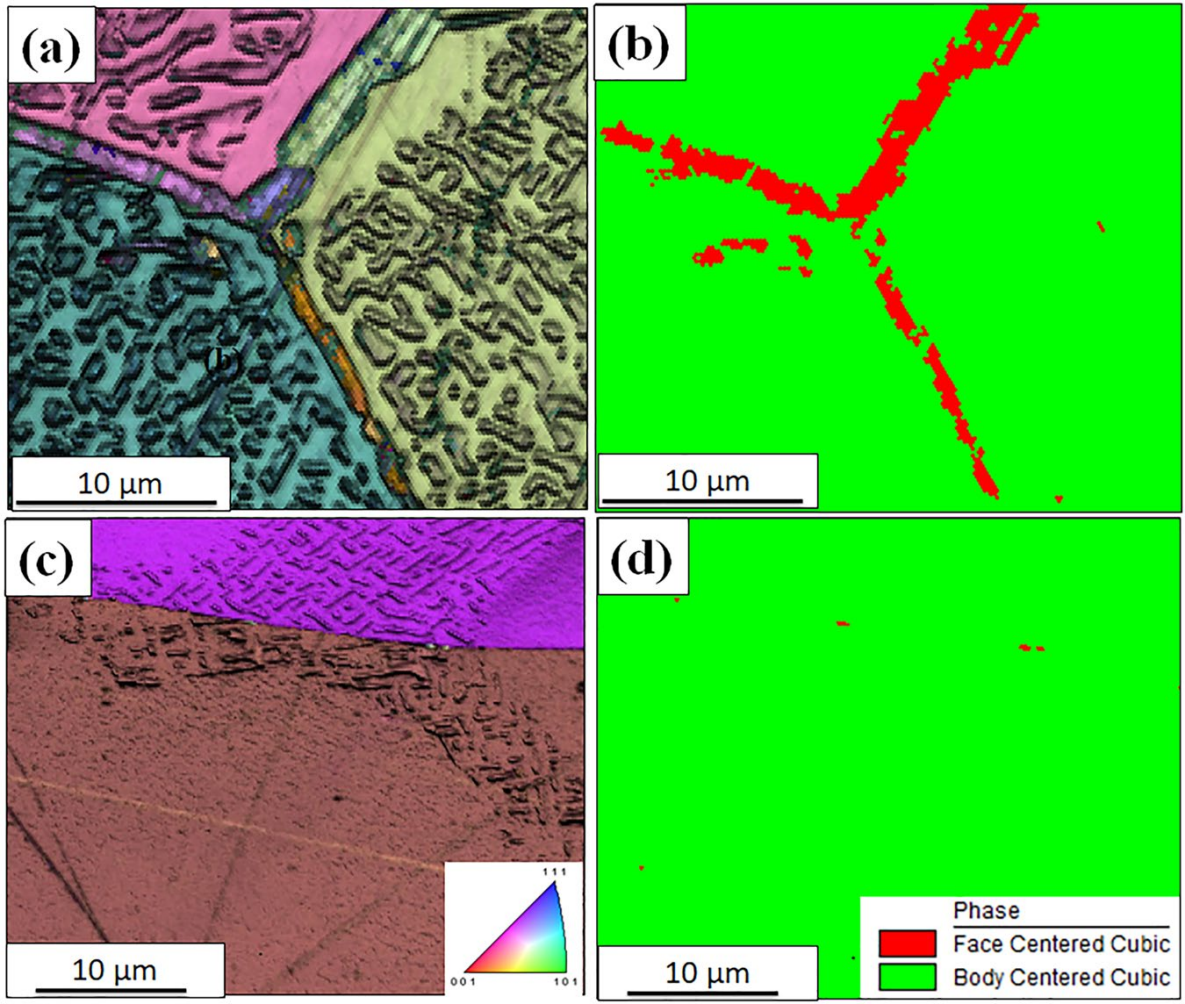
EBSD results: IPF maps of AlCo_0.5_Cr_0.5_FeNi annealed for 15 h at (**a**) 1000 °C, and (**c**) 600 °C. Corresponding phase maps are shown in (**b**) and (**d**), respectively.

Co was partially replaced by Cr in the case of the AlCo0.5Cr0.5FeNi alloy to investigate the influence of Cr addition on the microstructure. Figure 4(a) and (b) show back-scatter SEM images of the AlCo_0.5_Cr_0.5_FeNi specimens annealed for 15 h at 1000 °C, while Fig. 4(c) and (d) show SEM images from the 600 °C/15 h annealed sample. Both the heat treatment conditions resulted in the formation of a bright contrast faceted phase within a grey contrast matrix. Comparing the high magnification images from the two conditions, the 600 °C annealed sample (Fig. 4(d)) shows nano-scale precipitation in addition to the coarser micron-scale decomposition seen in both 600 °C and 1000 °C annealed samples. Note that the coarser precipitation of the bright phase is limited to the near-grain boundary region in the 600 °C sample (marked as BCC gen-1 in Fig. 4(d)) while it extended throughout the grains in the 1000 °C condition. Finer scale phase decomposition within the matrix is not visible in the case of the 1000 °C annealed sample. Based on these SEM observations it is apparent that phase separation occurred for both the annealing conditions in this alloy, unlike the AlCoFeNi alloy, which does not contain Cr. Hence, addition of Cr to AlCoFeNi substantially changes the microstructure by introducing a strong tendency of phase separation in the B2 phase to form a two-phase mixture. Apart from the intragranular precipitation/phase separation, there is clear evidence of a grain boundary phase forming in the case of the 1000 °C/15 h annealed sample, exhibiting a bright contrast (refer Fig. 4(a)). Therefore, orientation image microscopy (OIM) using an EBSD detector in the SEM, was performed to further examine the grain boundary regions in the two heat treated conditions of this alloy (Fig. 5). The inverse pole figure (IPF) maps, with the image quality map overlayed on top, from a representative region of the AlCo_0.5_Cr_0.5_FeNi alloy annealed for 15 h at 1000 °C and 600 °C are shown in Fig. 5(a) and (c), respectively. The corresponding phase maps with the FCC phase (in red) and the BCC phase (in green) are shown in Fig. 5(b) and (d) respectively. The coarse layer of the grain boundary phase was identified to be FCC in the case of the 1000 °C/15 h annealed sample, however within the grains, FCC was not observed. While intragranular decomposition is visible in this same sample, these regions were all indexed as a BCC phase in EBSD-OIM, indicating the absence of the FCC phase in these intragranular regions. This also indirectly indicates that the intragranular decomposition is a B2 + BCC, since both these phases are indistinguishable by EBSD-OIM.

Note that in the case of the 600 °C/15 h annealed AlCo_0.5_Cr_0.5_FeNi sample, there are a few small grain boundary FCC precipitates indexed in the phase map (right hand side of Fig. 5(b)), and these FCC precipitates do not form a continuous grain boundary layer, as observed in the case of the 1000 °C/15 h annealed sample. The composition of the different phases has also been analyzed using SEM-EDS. Supplementary Figure 1 shows the SEM-EDS maps of AlCo_0.5_Cr_0.5_FeNi specimens annealed at 1000 °C/15 h. The continuous matrix is rich in Al and Ni, while the discontinuous precipitates are enriched in Cr and Fe. SEM-EDS analysis shows minimal partitioning of Co between the B2 and BCC phases within the matrix grains. The grain boundary FCC phase is enriched in Cr, Fe, and Co while being depleted in Al and Ni.

#### TEM investigation of AlCo_0.5_Cr_0.5_FeNi (600 °C/15 h)

**Figure 6:**
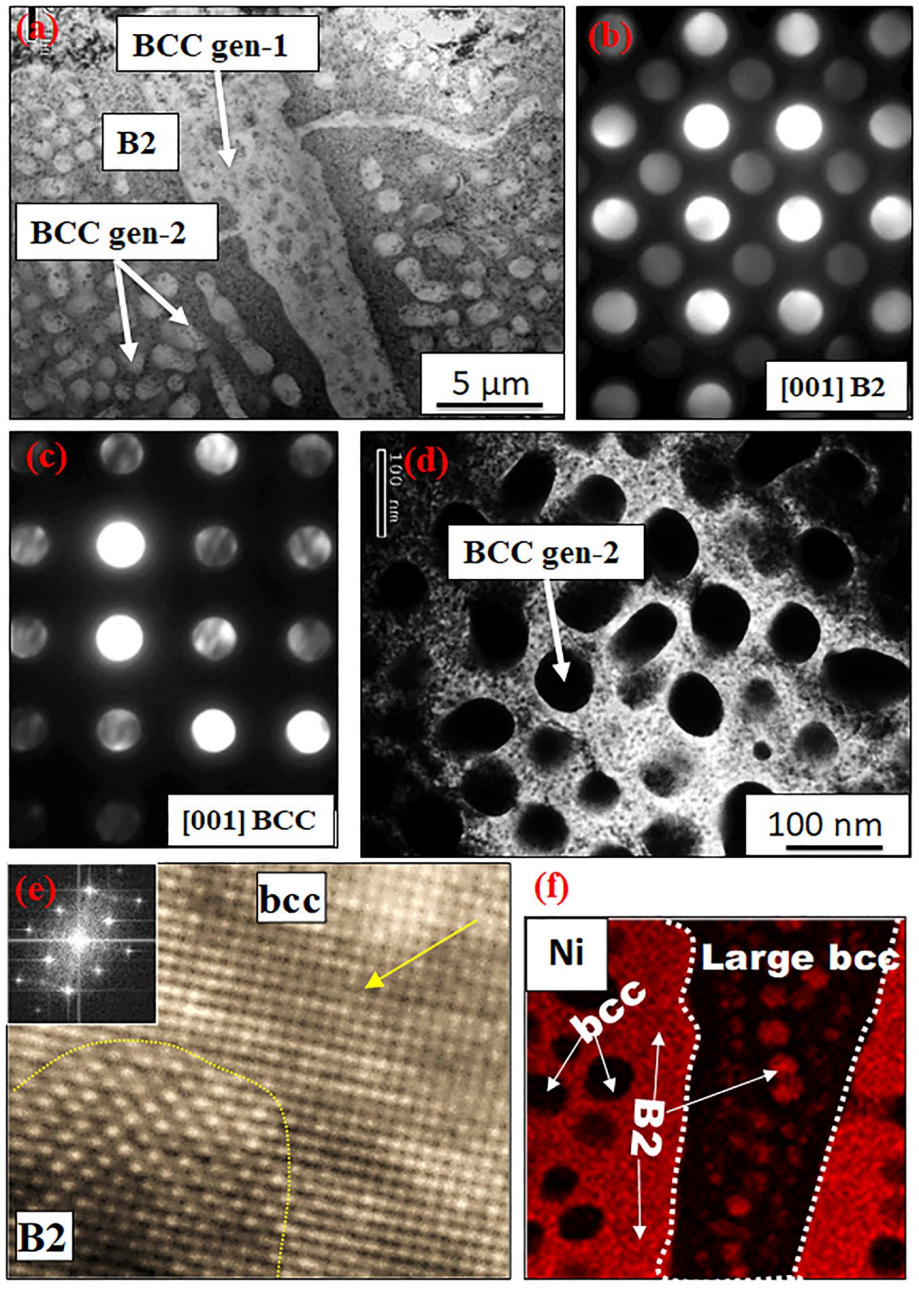
TEM results from AlCo_0.5_Cr_0.5_FeNi alloy annealed at 600 °C/15 h: (**a**) STEM image (**b**) SADP [001]_B2_ (**c**) SADP [001]_BCC_ (**d**) DFTEM image from a B2 spot (**e**) HRTEM image showing B2 and BCC phases and (**f**) STEM-EDS map showing Ni partitioning in between these phases.

Since the B2 + BCC decomposed microstructure after annealing the AlCo_0.5_Cr_0.5_FeNi alloy at 1000 °C/15 h was quite coarse (Fig. 4(a,b)), there was no requirement for a more detailed TEM investigation of this condition. In contrast, due to the highly refined scale of decomposition within the matrix grains in case of 600 °C/15 h annealed sample (as revealed by the SEM images shown in Fig. 4(d)), TEM investigations were carried out. Figure 6(a) shows a scanning TEM (STEM) image revealing phases exhibiting two different contrasts. Figure 6(b and c) show SADPs from a [001]_B2_ zone axis, captured from the phase exhibiting darker contrast (matrix) in Fig. 6(a), and from a [001]_BCC_ zone axis, captured from the phase exhibiting lighter contrast (precipitates) in Fig. 6(a), respectively. The {100} type superlattice spots in the [001] zone axis pattern shown in Fig. 6(b) confirms the B2 phase while the absence of these spots in Fig. 6(c) confirms the precipitates to be BCC. This proves that the microstructure is composed of a B2 matrix and BCC precipitates. Additionally, a dark-field image recorded from a {100} type B2 superlattice spot, clearly delineates the two phases with the dark disordered BCC regions (refer Fig. 6(d)). Additionally, the two different scales of the BCC phase have been marked as BCC gen-1 and BCC gen-2 in Fig. 6(a) and BCC gen-2 in Fig. 6(d). The high-resolution TEM image shown in Fig. 6(e) clearly reveals the continuity of the BCC lattice between the ordered B2 and disordered BCC phases. The yellow arrow in the fig points to {002} planes in the BCC and B2 phases, with the chemical ordering of atoms clearly visible in the region marked as B2. The high resolution TEM results clearly show the coherency between the B2 and BCC phases. Such coherency has been observed in other alloys, e.g., the Fe_55_(CrNiAl)_45_ alloys have two coherent phases, including the Ni-Al-rich B2 phase and the Fe-Cr-rich BCC phase^18^. Compositional analysis using TEM revealed more information about the composition of the phases. The HAADF-STEM EDS results (Fig. 6(f)) shows the elemental map of Ni atoms from a region containing both B2 and BCC phases. This map shows cubical/spherical precipitates of the BCC phase within the B2 matrix. The precipitation of B2 also occurs within the BCC lamella, as highlighted by the white arrow in the figure.

A region with high Cr concentration was also detected near the grain boundaries (refer Supplementary Figure 2). The SADP from this grain boundary phase can be consistently indexed as the [001] zone axis of the σ phase (Supplementary Figure 2(a)). The compositional analysis (Supplementary Figure 2(b)) showed that this grain boundary phase is the intermetallic sigma phase, which is often reported in Cr rich steels and HEAs^19^. The compositions of the phases B2, BCC (coarser gen-1 and finer scale gen-2) and sigma phases, based on the STEM-EDS measurements, are tabulated in Supplementary Table 1.

#### Atom Probe Tomography of AlCo_0.5_Cr_0.5_FeNi (600 °C/15 h)

**Figure 7:**
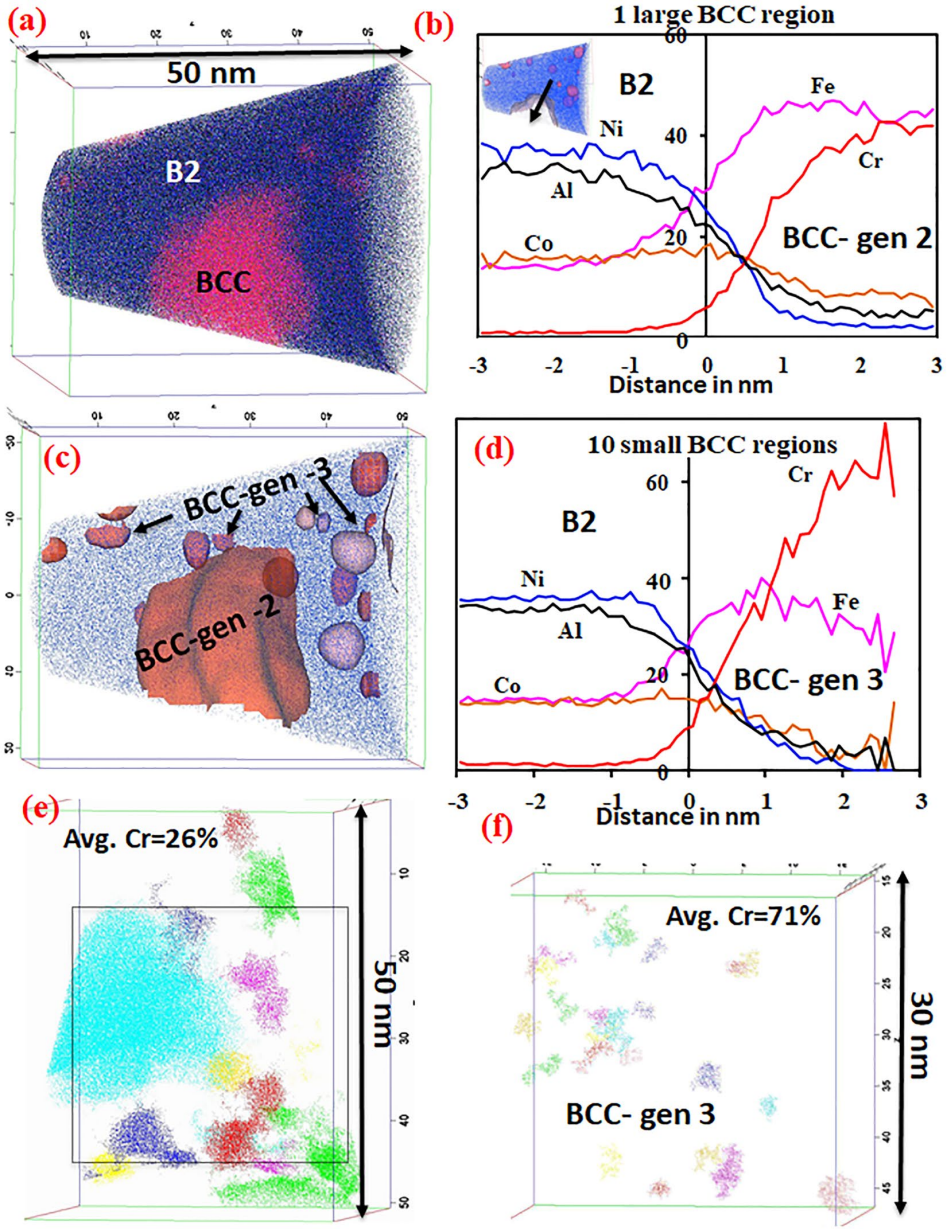
APT results from of AlCo_0.5_Cr_0.5_FeNi alloy annealed at 600 °C for 15 h. (**a**) APT reconstruction showing all ions (**b**) proximity histograms showing the compositional partitioning across the BCC generation-2 (the interphase is shown as the inset). (**c**) Reconstruction showing Ni (blue) ion and iso-surface is used to highlight the Cr rich regions. 10 random Cr rich region (small-generation 3) are used to generate a proximity histogram of the compositional partitioning across the interfaces is shown in (**d**). (**e**) Cluster analysis (dmax = 1, Nmin = 100) shows Cr rich regions with avg. 26% Cr (different colors are used to highlight different clusters) (**f**) Cluster analysis (dmax = 0.3, Nmin = 100, in a region marked by the square in (**e**) highlights Cr rich regions with avg. 71% Cr.

APT results from the AlCr_0.5_Co_0.5_FeNi alloy are shown in Fig. 7. The reconstruction in Fig. 7(a) shows the Ni ions in blue and Cr ions in red. The large Cr rich BCC region on the left of the reconstruction is marked as BCC gen-2 BCC and the fine (nanometre) scale Cr rich regions are marked as BCC gen-3 regions. This nomenclature is based on the precipitation sequence. The large micron-scale BCC lamellae, Figs 4(d) and 6(a), are the generation-1 BCC phase, precipitated in the early stages and consequently grown to a larger extent. Since, the APT samples were prepared in a site-specific manner from grain interiors, substantially away from the grain boundaries, the gen-1 BCC phase precipitates were not captured within the APT reconstructions, including the one shown in Fig. 7. A section of a gen-2 BCC precipitate has been captured within the 3D APT reconstruction shown in Fig. 7(a). A Cr-rich (Cr ~40 at%) iso-concentration surface (also referred to as isosurface in an abbreviated form) clearly delineates this gen-2 BCC precipitate, as shown in the inset in Fig. 7(c). Additionally, an even finer scale of Cr-rich precipitates is visible within the remaining B2 matrix in Fig. 7(c). These nanometer scale precipitates are not visible in the TEM images shown in Fig. 6, and are only visible in the APT reconstructions, revealing a new length scale of decomposition within this hierarchically decomposed microstructure. The compositional profile (using proxigram analysis) across generation-2 and generation-3 BCC precipitates are shown in Fig. 7(b) and (d), respectively. The finer scale generation-3 BCC phase contains ~30%Fe + 60%Cr, compared to the generation-2 BCC which contains ~45%Fe + 40%Cr.

Furthermore, the phase separation tendency into solute lean and solute rich regions can be captured using cluster analysis tools applied to the APT data. The data-mining technique of cluster finding was employed; the step-by-step procedure is described in the IVAS™ 3.6.8 manual. Cluster analysis, in essence, identifies for a chosen atomic species, where the distance (d_max_) between two atoms is smaller compared to the rest of the dataset. The values of d_max_ and N_min_ (minimum number of atoms in a cluster) were optimized to maximize the probability to detect any compositional clustering. With further data analysis the three-dimensional region with interatomic distances equal to or smaller than optimized valued of d_max_ and N_min_ (that yielded the biggest count of non-random clusters) were extracted from the data set. Using d_max_ = 1 (optimized using cluster counter distribution analysis on the APT data, refer Supplementary Figure 3) and N_min_ = 100 ions, 14 clusters, with an average total atom/ion count of ~16000 ions and Cr concentration ~26%, were detected (shown in Fig. 7(e)). The Cr concentration ranges from 16%-42%. With d_max_ = 0.3 and N_min_ = 100 ions (in a region marked by the square in Fig. 7(e)), 25 clusters with average total ion count of 332 ions and Cr concertation of 71% were detected. The Cr concentration ranges from 62–74% in these clusters (shown in Fig. 7(f)), corresponding to the BCC-gen3 precipitates in this microstructure. Therefore, this cluster analysis further supports the presence of different generations of Cr rich BCC precipitates within the B2 phase.

#### Thermodynamic Modeling of AlCo_x_Cr_1−x_FeNi

**Figure 8:**
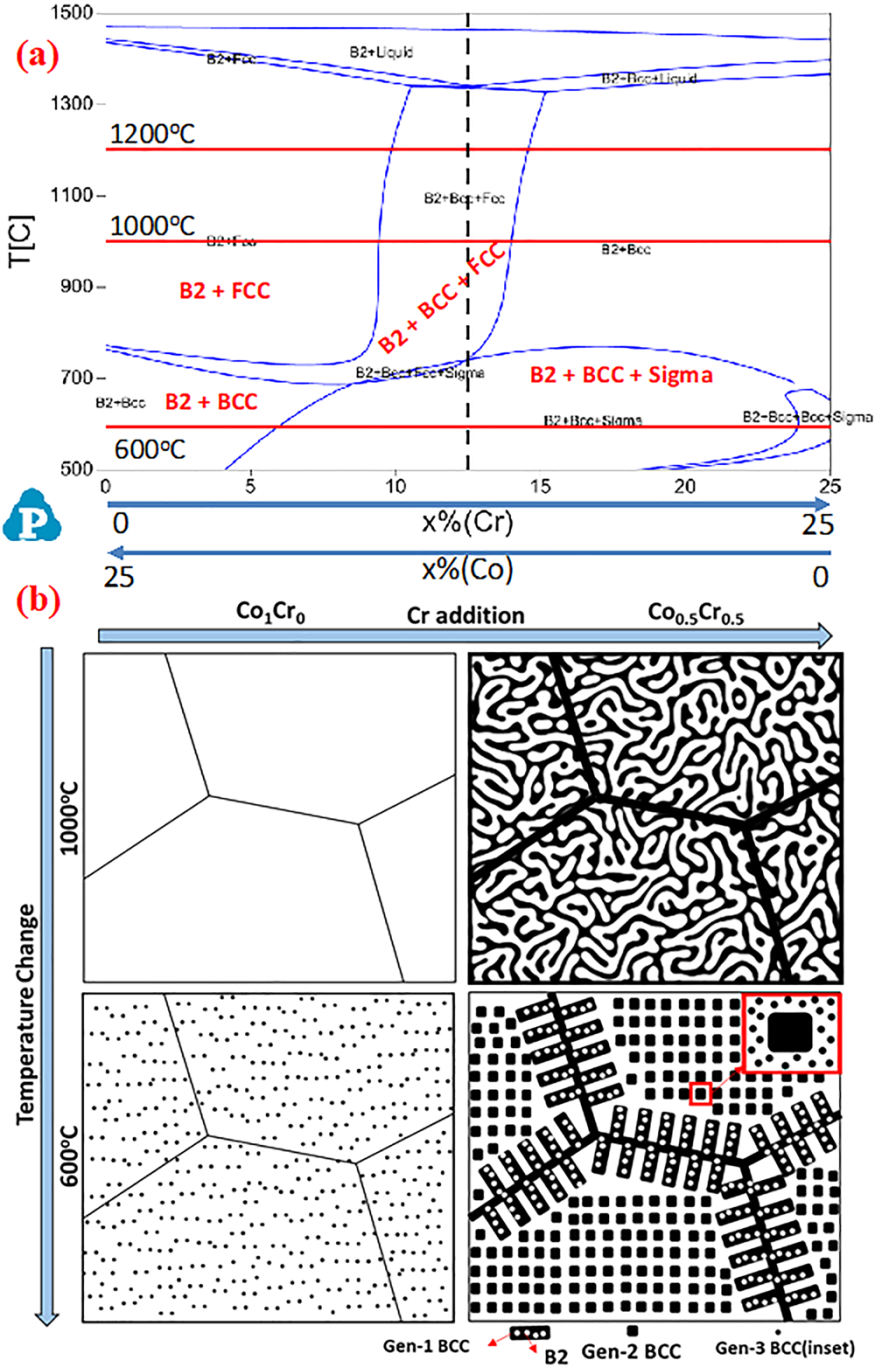
(**a**) Isopleth of AlCo_1−x_Cr_x_FeNi system computed using PANDAT using the PanHEA database. (**b**) A schematic diagram showing the phase transformations based on effect of annealing and effect compositional change (Cr addition).

The experimentally observed microstructures and the implied phase stability were validated by thermodynamic calculations using the CALPHAD approach within the framework of the PANDAT software^20^. Figure 8(a) shows a computed isopleth of the change in phase stability as a function of Cr content and temperature in these alloys. The predicted equilibrium phases for the AlCoFeNi alloy, on the extreme left of this isopleth, at 1000 °C consists of the B2 and FCC phases while at 600 °C consists of the B2 and BCC phases. On the other hand, in the case of the AlCr0.5Co0.5FeNi alloy, which lies at the center of the isopleth, the equilibrium phases at 1000 °C are the B2, BCC and FCC phases while at 600 °C the equilibrium phases are the B2, BCC and sigma phases. Our experimental investigations match very well with the thermodynamic predictions, except for the FCC phase predicted in AlCoFeNi alloy at 1000 °C. Note that there is likely to be a high thermodynamic nucleation barrier for the precipitation of the FCC phase within the B2 matrix owing to the high interfacial energy between these two phases^19^. Consequently, the kinetics of precipitation could be extremely sluggish, and FCC precipitation could be limited solely to high angle grain boundaries which are high energy sites. However, in the experimental observations, there was no conclusive FCC formation observed in case of the AlCoFeNi 1000 °C/15 h annealed sample.

### Microhardness and Magnetic Properties

The Vickers microhardness of both the AlCoFeNi and AlCo_0.5_Cr_0.5_FeNi alloy for both heat-treated conditions (600 °C and 1000 °C annealing) have been shown in the bar chart in Supplementary Figure 3 and the values have also been listed in Table 1. The lowest microhardness value of 406 VHN was observed for the AlCoFeNi alloy annealed at 1000 °C, while the highest microhardness value of 475 VHN was observed in case of the AlCo_0.5_Cr_0.5_FeNi alloy annealed at 600 °C. These observations can be qualitatively rationalized based on the fact that in case of the former, a single B2 phase is present in the 1000 °C annealed condition of the AlCoFeNi alloy, while in case of the latter a multi-scale hierarchical B2 + BCC decomposed microstructure was observed in case of the 600 °C annealed condition of the AlCo_0.5_Cr_0.5_FeNi alloy. Consequently, the single phase B2 microstructure has a lower hardness as compared to the hierarchically decomposed B2 + BCC microstructure. Interestingly, the 600 °C annealed condition of the AlCoFeNi alloy also exhibits a very high microhardness of 474 VHN, despite the fact that only an early stage of phase decomposition into B2 + BCC was exhibited by this sample.

**Table 1:** Saturation magnetization and coercivity values for the AlCoFeNi and AlCo_0.5_Cr_0.5_FeNi alloys for the different heat-treated conditions.

Alloy	Heat treatment	Ms (emu/g)	Hc (Oe)
AlCoFeNi	600 °C/15 h	99.8	14.5
AlCoFeNi	1000 °C/15 h	111.6	4.3
AlCo0.5Cr0.5FeNi	600 °C/15 h	46.2	95.6
AlCo0.5Cr0.5FeNi	1000 °C/15 h	42.2	39.4

The saturation magnetization (*M*_*S*_) and coercivity (*H*_*c*_) for the four different samples analyzed in the present study have been listed in Table 1. The *M*_*S*_ of AlCoFeNi is substantially higher compared to that of the AlCo_0.5_Cr_0.5_FeNi alloy for both heat-treated conditions. While the magnetization is primarily determined by composition and the crystal structure, the coercivity can be affected by impurities, deformation, grain size and phase decomposition^21^. The coercivity (*H*_*c*_) decreased with increasing annealing temperature from 15 Oe for sample aged at 600 °C/15 h, exhibiting nanoscale phase separation, to 4 Oe for samples aged at 1000 °C/15 h, exhibiting a homogeneous undecomposed B2 matrix. A similar observation has been reported in the literature, where the coercivity of the AlCoCrCuFeNi HEA annealed at 1000 °C/2 h decreases by 30 Oe (from 45 Oe to 15 Oe) compared to as-cast alloys^22^. The AlCoFeNi HEA investigated in the present study exhibits higher saturation magnetization and lower coercivity than other soft ferrite magnetic materials, e.g., MnZn ferrite, Cu-Zn-Ti ferrite^23,24^, as revealed in Supplementary Table 2.

The saturation magnetization of the AlCo_0.5_Cr_0.5_FeNi HEA reduces by more than 50% compared to samples with no Cr (AlCoFeNi). It has been previously reported earlier that *M*_*S*_ falls linearly with increasing Cr content^13,25,26^. This is mainly due to the antiferromagnetic nature of Cr. Additionally, the coercivity of the Cr containing alloy was higher than the alloy with no Cr. The *M*_*S*_ and *H*_*C*_ of AlCo_0.5_Cr_0.5_FeNi decreases with increasing annealing temperature. It has been previously reported that magnetic hardening of Fe–Co–Cr alloys is associated with the formation of a two-phase microstructure at the nanometer scale; firstly, the Fe–Co rich ferromagnetic phase, and secondly, the Cr rich antiferromagnetic phase. The relationship between the decomposition of the B2 phase into a two-phase B2 + BCC microstructure and the resulting coercivity is not well understood in these alloy systems and requires further detailed investigation.

Supplementary Table 2 shows a comparison of the magnetic properties (*M*_*S*_ and *H*_*C*_) of our current work with previous reports^21,22,27–34^ in the literature on HEAs. It can be concluded from Supplementary Table 2 that the current HEAs, especially AlCoFeNi, exhibit more favorable magnetic properties with the best combination of saturation magnetization (112 emu/g) and coercivity (4 Oe), compared to all the other recently reported HEAs. AlCo_0.5_Cr_0.5_FeNi showed relatively low value of *M*_*S*_ and reasonably low coercivity due to the addition of anti-ferromagnetic Cr to this alloy. However, though Cr is detrimental to the soft magnetic properties, this alloying addition has beneficial effects in terms of other properties such as corrosion resistance^2,35,36^.

### Effect of Cr substitution on hierarchical phase decomposition in AlCo_x_Cr_1−x_FeNi alloys

The substitution of Co by Cr in these AlCo_x_Cr_1−x_FeNi alloys has rather interesting consequences on the phase stability. Without Cr addition, AlFeCoNi exhibits a single homogeneous B2 phase at higher temperatures (annealed at 1000 °C) and exhibits a weak tendency for phase separation into B2 + BCC phases on annealing at lower temperatures, such as at 600 °C for 15 h. The observed experimental results are consistent with the predictions of solution thermodynamic models, such as PANDAT using the PanHEA database^20^ in this case. Addition of Cr strongly enhances the tendency of the B2 phase to decompose into a B2 + BCC mixture, as observed from the experimental results from the AlCo_0.5_Cr_0.5_FeNi alloy. Even at higher temperatures, such as 1000 °C, the B2 + BCC phase separation is evident (refer to Fig. 4(a,b)). The PANDAT computations also indicate that the B2 + BCC + FCC phase field, for this composition, extends to a very high temperature of ~1400 °C (refer to isopleth shown in Fig. 8). The strong tendency of Cr to introduce phase separation in the B2 phase can possibly be attributed to a positive enthalpy of mixing of Fe-Cr, Co-Cr and Ni-Cr systems^37^, leading to miscibility gaps observed in these binary systems and a tendency for phase separation. However, it should be noted that Al-Cr binary system has a propensity for compound formation due to negative enthalpy of mixing^37^. In all, it appears that the phase separation tendency in the current HEA is introduced by Cr. The details of the thermodynamic basis underlying the influence of Cr on phase separation in these complex concentrated multi-component alloys requires further analysis beyond the scope of the present paper. Nevertheless, the importance of Cr in promoting phase separation in these HEAs needs to be recognized. The length scale of this phase separation appears to be dependent on the annealing temperature. A coarse length scale of decomposition is observed in the case of the AlCo_0.5_Cr_0.5_FeNi alloy after 1000 °C/15 hrs annealing (decomposition in the micron length scale), while a substantially refined length scale of decomposition is observed in the case of the same alloy after annealing at 600 °C/15 h. Additionally, the 600 °C/15 h annealed AlCo_0.5_Cr_0.5_FeNi alloy exhibited multiple length scales of B2 + BCC decomposition, ranging from the majority of the matrix exhibiting a decomposition length scale of ~50–100 nm (refer to Fig. 6(a) and (d)) to a decomposition length scale ~3–5 nm revealed by atom probe tomography (APT) within the B2 matrix (refer to Fig. 7).

The influence of Cr substitution for Co, and temperature, on phase decomposition is these alloys has been schematically described in Fig. 8(b). While the AlCoFeNi alloy exhibits a polycrystalline single phase B2 microstructure at 1000 °C, represented by the top left schematic in Fig. 8(b), reducing the temperature to 600 °C induces the early stages of phase separation into B2 + BCC, as represented by the schematic on the bottom left. A much stronger influence on the phase separation is induced by the addition of Cr to AlCoFeNi. Thus, in case of the AlCo_0.5_Cr_0.5_FeNi alloy, at 1000 °C there is a coarse decomposition into B2 + BCC phases. Reducing the temperature to 600 °C, results in a very complex microstructure exhibiting phase separation over multiple length scales, as depicted in the schematic on the bottom right in Fig. 8(b). There is a coarse phase separation into B2 + BCC, with coarse BCC lamellae along the grain boundaries. A finer scale phase separation is observed within the grains, with near-cuboidal BCC precipitates homogeneously distributed within the B2 matrix. The inset in this schematic shows that there is an even finer scale of phase separation within the B2 matrix, resulting in nanometer scale BCC clusters.

## Conclusions

The microstructure and magnetic properties of AlCoFeNi and AlCo_0.5_Cr_0.5_FeNi HEAs for two different annealing conditions were investigated. The focus was on probing the influence of changing the Co/Cr ratio (replacing Co by Cr) and temperature, on the phase decomposition and its consequent impact on magnetic properties and microhardness, in these HEAs. While the AlCoFeNi alloy exhibits a compositionally homogeneous single B2 phase on annealing at higher temperatures (1000 °C), it tends to decompose into a two-phase mixture of B2 + BCC on annealing at lower temperatures (600 °C). Substituting half of the Co by Cr, resulting in the AlCo_0.5_Cr_0.5_FeNi HEA, strongly enhances the tendency of phase separation in these alloys to form a two-phase B2 + BCC mixture for all annealing temperatures. Furthermore, annealing at 600 °C, leads to a hierarchical microstructure with multiple length scales of decomposition ranging from few nanometers to microns. This decomposition leads to a substantially higher microhardness coupled with a decrease in saturation magnetization (*M*_*S*_) and increase in coercivity (*H*_*C*_). Overall, the AlFeCoNi alloy exhibited the best combination of soft magnetic properties, with saturation magnetization (*M*_*S*_) and coercivity (*H*_*C*_) values of 112 emu/g and 4 Oe, after annealing at 1000 °C for 15 h, respectively.

## Electronic supplementary material

Supplementary information
