# Supplementary material for: Influence of Cr Substitution and Temperature on Hierarchical Phase Decomposition in the AlCoFeNi High Entropy Alloy
Source: Sci Rep. 2018 Oct 22;8:15578. doi: 10.1038/s41598-018-33922-w (PMC6197204; doi:10.1038/s41598-018-33922-w)
Supplement: Supplementary file 1 — Supplementary information [file 41598_2018_33922_MOESM1_ESM.pdf]

Influence of Cr Substitution and Temperature on Hierarchical Phase Decomposition in the AlCoFeNi High Entropy Alloy

V. Chaudhary[1#], B. Gwalani[2#], V. Soni[2], R.V. Ramanujan[1] and R. Banerjee[2*]

[1]School of Materials Science and Engineering, Nanyang Technological University, Singapore 639798, Singapore
[2]Department of Materials Science and Engineering, University of North Texas, Denton, TX 76201, USA
*Email: Rajarshi.Banerjee@unt.edu

# Equal contributing authors

Supplementary Figures:

Supplementary Figure 1: SEM-EDS maps from $AlCo_{0.5}Cr_{0.5}FeNi$ alloy annealed for 15 h at 1000 ^{0}C.

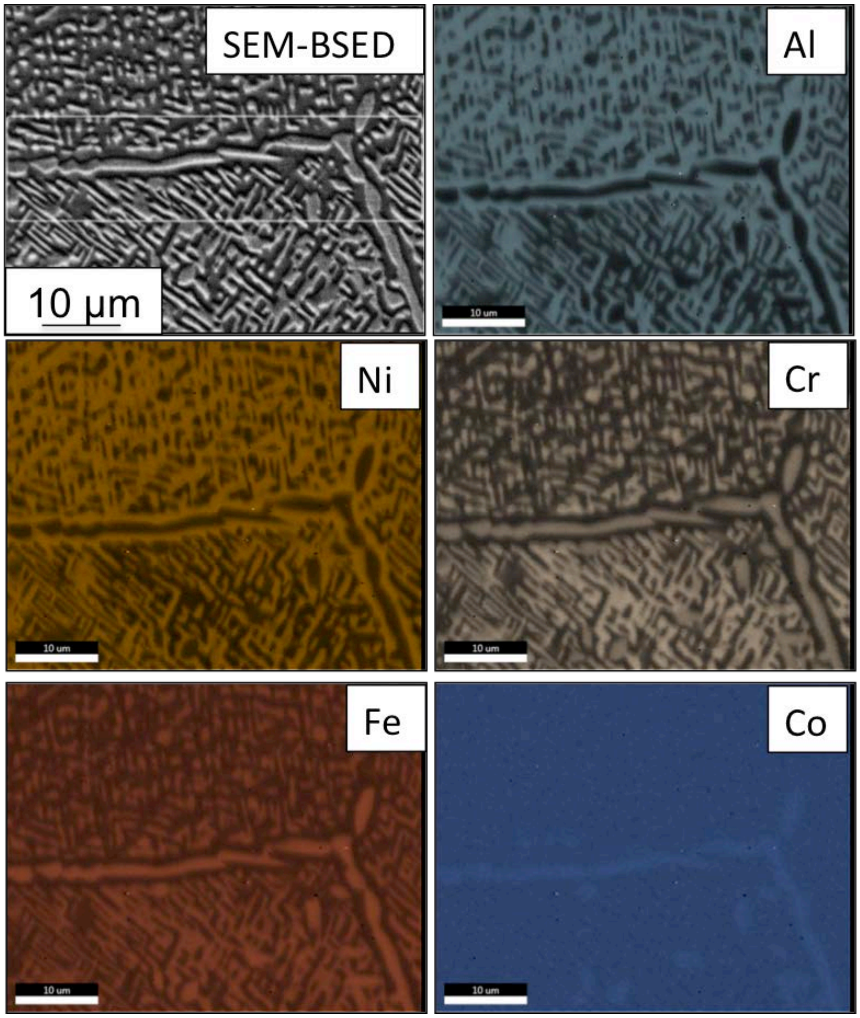

Supplementary Figure 2: TEM results from the grain boundary region in AlCo0.5Cr0.5FeNi alloy annealed for 15 h at 600 oC, (a) SADP from sigma phase (b) STEM-EDS map showing the partitioning of Cr.

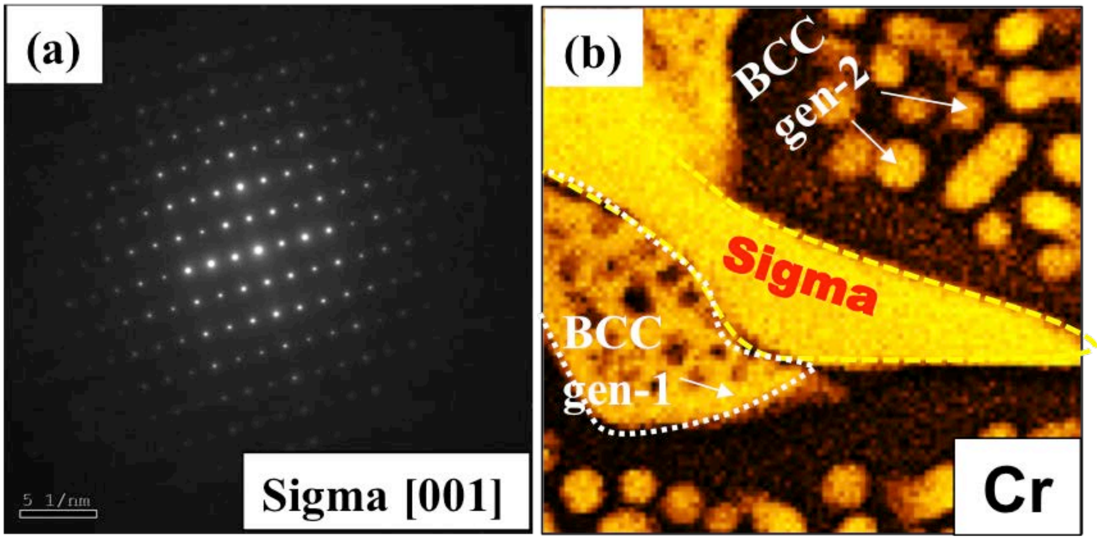

Supplementary Figure 3: Microhardness of the AlCoFeNi and AlCo0.5Cr0.5FeNi alloys after annealing treatments at 600°C and 1000°C.

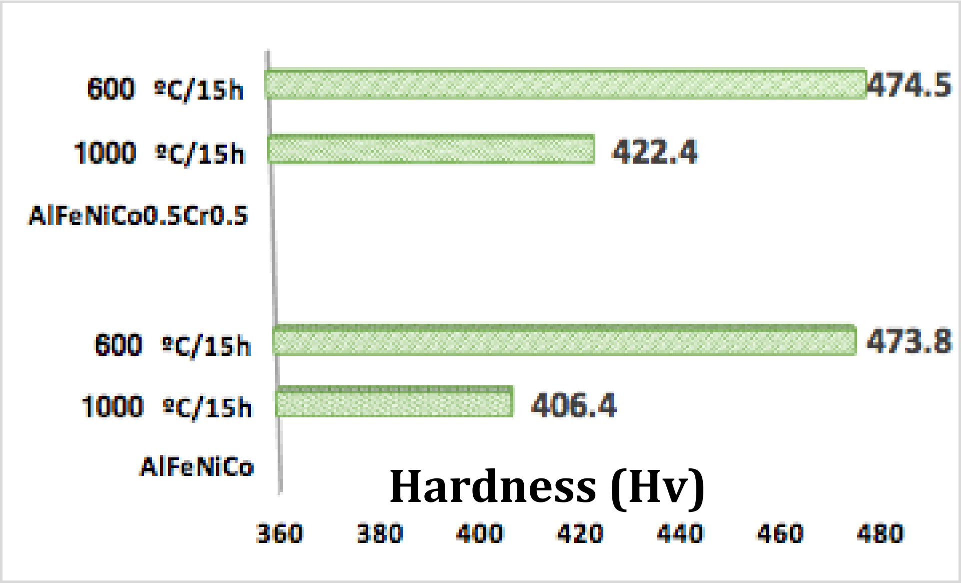

Supplementary Figure 4: Cluster count distribution analysis on the APT data from the AlCo0.5Cr0.5FeNi alloy after annealing treatments at 600°C.

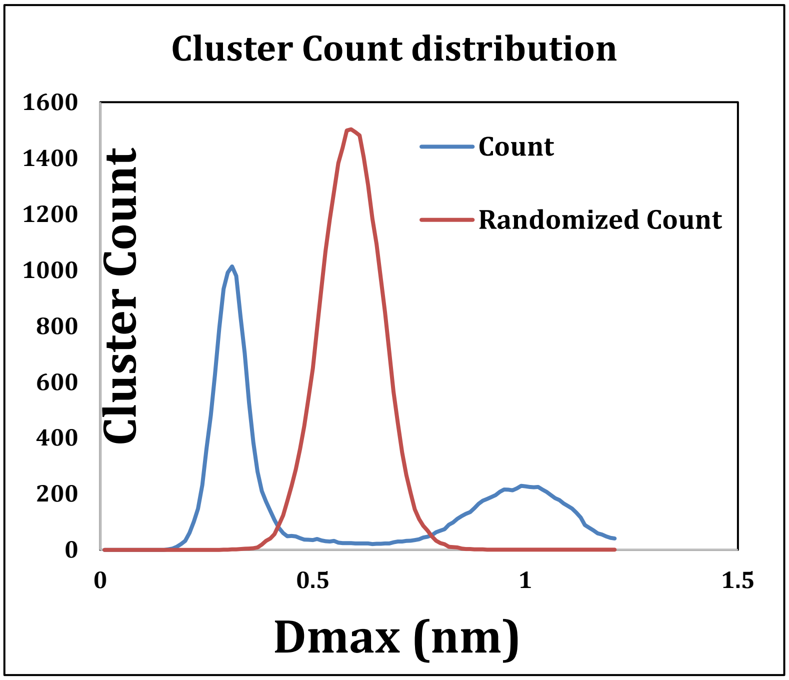

Supplementary Tables:

Supplementary Table 1: STEM-EDS composition (at%) of the phases present in $AlCo_{0.5}Cr_{0.5}FeNi$ alloy after 600 °C/15 h annealing.

**Phases→**	**Sigma**		**B2**		**Gen-1 BCC**		**Gen-2 BCC**	
**Elements**	**At%**	**Uncert.%**	**At%**	**Uncert.%**	**At%**	**Uncert.%**	**At%**	**Uncert.%**
**Al(k)**	1.05	0.01	28.23	0.2	10.51	0.11	11.12	0.14
**Cr(k)**	43.67	0.14	5.52	0.13	27.52	0.26	26.2	0.3
**Fe(k)**	40.3	0.16	17.77	0.26	38.4	0.34	38.81	0.41
**Co(k)**	12.61	0.09	14.36	0.24	12.84	0.21	11.6	0.25
**Ni(k)**	2.35	0.04	34.09	0.36	10.71	0.19	12.25	0.24

Supplementary Table 2: Comparison of saturation magnetization (Ms) and coercivity (HC) of high entropy alloys.

**HEAs**	**Phase(s)**	**Ms(emu/g)**	**Hc (Oe)**	**Ref**
AlCoCrCuFeNi	-	16-38	15-45	[22]
AlCoCrCuFeNi	-	44-48	44-264	[28]
AlCoCrFeNi	BCC	64	51.4	[29]
AlCoCuFeNi	BCC + FCC	84	162	[30]
$AlCoCrFeNiNb_{0.25}$	BCC + Laves	25	94	[29]

$Al_{0.25}CoFeMn_{0.25}Ni$	FCC	101	3.5	[21]
CoCrFeMnNi	FCC	1.39	135	[31]
CoFeMnNiSn	L21 + BCC	80	43	[31]
CoFeGaMnNi	BCC + FCC	80	11.5	[31]
AlCoFeMnNi	B2+BCC	148	7.9	[31]
$(AlCu)_{0.8}FeCoGa_xNi$ ($0 \leq x \leq 0.08$)	BCC+FCC	79-83	4.5-8.6	[32]
$Al_{1.25}FeCoCrNi$	BCC	43	18	[33]
$CoCrFeNiPd_{1-2}$	FCC	33-34	-	[34]
$Al_2CoCrFeNi$	BCC	13-18	-	[34]
AlCoFeNi		100-112	4-15	This work
$AlCo_{0.5}Cr_{0.5}FeNi$		42-46	39-96	This work

Supplementary Figure captions:

Supplementary Fig. 1. SEM-EDS maps from $AlCo_{0.5}Cr_{0.5}FeNi$ alloy annealed for 15 h at 1000 ^oC.

Supplementary Fig. 2. TEM results from the grain boundary region in $AlCo_{0.5}Cr_{0.5}FeNi$ alloy annealed for 15 h at 600 ^oC, (a) SADP from sigma phase (b) STEM-EDS map showing the partitioning of Cr.

Supplementary Fig. 3. Microhardness of the AlCoFeNi and $AlCo_{0.5}Cr_{0.5}FeNi$ alloys after annealing treatments at 600°C and 1000°C.

Supplementary Fig. 4. Cluster count distribution analysis on the APT data from the $AlCo_{0.5}Cr_{0.5}FeNi$ alloy after annealing treatments at 600°C.

Supplementary Table captions:

Supplementary Table 1: STEM-EDS composition (at %) of the phases present in $AlCo_{0.5}Cr_{0.5}FeNi$ alloy after 600°C/15hrs annealing.

Supplementary Table 2. Comparison of saturation magnetization (*Ms*) and coercivity (H_C) of high entropy alloys.
